# Effects of orthopaedic treatments upon 3D radiologic morphologies and equilibrated postures in adolescent idiopathic scoliosis

**DOI:** 10.1186/1748-7161-9-S1-P4

**Published:** 2014-12-04

**Authors:** Julie Deceuninck, Jean-Claude Bernard

**Affiliations:** 1 CMCR des Massues, Lyon, France

## Introduction

In case of scoliosis, spinal deformations are measured upon frontal radiographs. However, these deformations are developed in 3D space. Recent studies have been proposed to access to 3D features of spine and pelvis in upstanding patients.

## Material and method

Geometric structures of spinal curves of scoliotic patients have been identified. They show plane regions where spinal curves are purely flexed. These regions are linked together by zones of connection, where abduction and axial rotation components, are concentrated. Spine and pelvis of each patient radiographed in upstanding may be synthetized by two rows of indices: one row describing the morphology, the second one illustrating the postural stability. The present study is dedicated to effects upon 3D back morphology and postural stability of orthopaedic treatments exerted to a same patient.

## Results

Orthopaedic treatments may significantly change the patient back morphology. The series presents effects of orthopaedic treatments when results of the 3D analysis of morphology and postural stability in initial situation were unknown for the technical operator in charge of cast or brace design. Technique and preliminary results are presently analyzed by clinical people: rehabilitation doctors and physiotherapists.

